# Retrospective real-world pilot data on transcranial pulse stimulation in mild to severe Alzheimer's patients

**DOI:** 10.3389/fneur.2022.948204

**Published:** 2022-09-14

**Authors:** Celine Cont, Nathalie Stute, Anastasia Galli, Christina Schulte, Kazimierz Logmin, Carlos Trenado, Lars Wojtecki

**Affiliations:** ^1^Departmemt of Neurology and Neurorehabilitation, Hospital Zum Heiligen Geist, Academic Teaching Hospital of the Heinrich-Heine-University Duesseldorf, Kempen, Germany; ^2^Institute of Clinical Neuroscience and Medical Psychology, Medical Faculty, Heinrich-Heine-University Düsseldorf, Düsseldorf, Germany; ^3^Max Planck Institute for Empirical Aesthetics, Frankfurt am Main, Germany

**Keywords:** neuromodulation, transcranial pulse stimulation, Alzheimer's disease (AD), dementia, real-world data

## Abstract

**Introduction:**

Transcranial pulse stimulation (TPS) is a non-invasive neuromodulation therapy that uses short, repetitive shockwaves through a neuro-navigated device. Current research suggests that these pulses lead to a wide range of vascular, metabolic, and neurotrophic changes. This relatively new CE-marked treatment provided first promising results in a clinical pilot study for improving cognition in mild-to-moderate Alzheimer's. Data from other centers is lacking, so here we analyzed safety and pilot real-world short-term results of TPS from the first center in Germany. To gain information about effects in different stages, patients with not only mild but also moderate-to-severe Alzheimer's were analyzed.

**Methods:**

A total of 11 patients were retrospectively examined for cognitive and emotional function before and after the first stimulation series. The effect was assessed using several neuropsychological tests [Alzheimer's Disease Assessment Scale (ADAS), including the ADAS cognitive score (ADAS Cog) and ADAS affective scores, Mini-Mental Status Examination (MMSE), and Montreal Cognitive Assessment (MoCA)] including in comparison between the groups of mild-to-severe patients. Moreover, subjective improvement of symptom severity, potential effects on depressive symptoms, and side effects were analyzed using Numeric Rating Scales (NRS).

**Results:**

Side effects were rare (in 4% of sessions) with moderate subjective severity and only transient. Patients significantly improved in the ADAS and ADAS Cog, while there was no significant effect in MMSE and MoCA. Patients' self-reported symptom severity improved significantly. The depressive symptoms measured in an ADAS subscale also improved significantly. Statistical data analyses revealed no significant correlation of clinical improvement with baseline symptom severity.

**Conclusion:**

TPS might be a safe and promising add-on therapy for Alzheimer's, even for moderate-to-severe patients. More research on long-term effects in patients as well as studies with sham control groups is needed. Moreover, translational research on the mechanisms of action and effects on cerebral network physiology will be needed to understand this new neuromodulation technique.

## Introduction

The most common type of dementia is Alzheimer's disease (AD), which is defined as a progressive neurodegenerative disease characterized by plaques and neurofibrillary tangles (1). Symptoms of this dementia are characterized mainly by a decline in memory and independence in personal daily activities. Around 50 million patients suffer from this disease, and no treatment is available to prevent or cure AD. Two types of symptomatic drugs are used, including cholinesterase inhibitors and antagonists to N-methyl-d-aspartate (1). One newly FDA-approved drug, called Aducanumab, is one of the first approved medications to target the possible cause of AD. This monoclonal antibody clears out the plaque of amyloid-ß (2). However, more research is needed. As a non-pharmacological treatment, non-invasive brain stimulation (NiBS) has already shown encouraging preliminary results as it integrates the multilevel biological and neurophysiological complexity of AD (3–5). For AD, different brain stimulation techniques are already used: transcranial magnetic stimulation (TMS) as well as electrical stimulation using transcranial direct current stimulation (tDCS) and transcranial alternating current stimulation (tACS), the latter with a possible amyloid-clearance effect using gamma frequencies (6). Recent reviews suggest the use of NiBS in AD as promising, yet it should be used in addition to multidisciplinary therapies (5).

One relatively newly CE-marked therapy for AD is transcranial pulse stimulation (TPS) (7). TPS might have some advantages compared to other neuromodulation devices: it is applied highly focal and is possibly not restricted to superficial layers of the brain and, therefore, stimulates up to 8 cm in depth. This non-invasive neuromodulation therapy uses short, single pulses of mechanical waves called shockwaves. The characteristics of shock repetitive waves are pulses that each last about 1 μs. In contrast to ultrasound, the pulse is followed by a tensile wave with a relieving effect of lower amplitude, which lasts for about 4–5 μs. Subsequently, the result is a reciprocal effect with high pressure and low tension, emerging due to the asymmetrical pulse validating both momentums, which do not compensate for each other. The focal energy deposition was tested for its practicability with rats, human skulls, and brain specimens (7). The results of the stimulation of mechanosensitive ion channels manifest themselves in increased metabolism, angiogenesis, and anti-inflammatory effects caused by the release of nitric oxide in the treated areas (7). The stimulation affects vascular growth factors (VEGF), neurogenesis (eNGF and GF-2), and brain-derived neurotrophic factors (BDNF) (8). The first evidence for beneficial clinical effects after a series of six TPS sessions in an uncontrolled pilot study with 35 AD patients discovered an effect on cognitive performance after TPS treatment (7). The cognitive effect was measured using the CERAD test, which significantly improved after treatment, with an increase of total points of about 10.5%. This effect lasted up to a 3-month follow-up period. Additionally, a significant improvement of depressive symptoms after 2–4 weeks of TPS treatment was reported, which suggests TPS as an add-on therapy for depression in AD (10).

However, besides in healthy subjects (9), no AD placebo-controlled trial has been published for TPS. Furthermore, there is a lack of information about the real-world applicability, safety, and effects of other centers outside the pioneer center in Vienna. Therefore, in this paper, we provide a pilot retrospective analysis of the feasibility, safety, and short-term effect of TPS on the cognitive and emotional performance of 11 patients with AD as the first center in Germany. To date, TPS is recommended for mild-to-moderate AD. Furthermore, we investigated patients with severe forms. Specific hypotheses were as follows:

I) TPS is safe and generally well tolerated.
II) TPS improves subjective symptom severity in cognition and mood.
III) TPS shows positive short-term effects on cognitive functions assessed in the objectives test.
IV) TPS is effective in patients with mild, moderate, and severe AD.

## Methods

### Patients

A consecutive number of 11 TPS-treated patients with Alzheimer's disease from the Department of Neurology and Neurorehabilitation at Hospital zum Heiligen Geist in Kempen, Germany, were examined (nine men, two women, age range 59–77 years, *M* = 69.82). Inclusion and exclusion criteria for TPS treatment were based on clinical evaluations, MRI, CSF, and EEG. The inclusion criteria was at least Alzheimer's clinical syndrome, which was defined in a gradual progressive change in memory function (using the MMSE as screening tool for severity score) and impairment of activity of daily living for more than 6 months. *In vivo* evidence from CSF and/or MRI scans and/or PET was used for the NIA-AA criteria, which categorizes the underlying pathological processes using biomarkers (11). These biomarkers are grouped into ß amyloid deposition, pathological tau, and neurodegeneration [AT(N)], which can be detected in imaging and biofluids. The biomarker category is shown in Table 1. A total of eight patients were defined as Alzheimer's continuum, seven of them with Alzheimer's disease (AD) and one with Alzheimer's disease and concomitant suspected non-Alzheimer's pathological change. Two patients were simply defined as having Alzheimer's clinical syndrome due to a lack of biomarker and one as having Alzheimer's clinical syndrome with non-Alzheimer's pathological change.

**Table 1:** Demographics of the patients.

**ID**	**Age**	**Sex**	**Cognitive impairment**	**Biomarker category / diagnosis**
1	76	M	Mild	A+T+(N)+ / AD
2	74	M	Severe	A+T+(N)+ / AD
3	77	M	Moderate	Alzheimer's clinical syndrome without biomarkers tested
4	59	M	Moderate	A+T-(N)+^a^
5	60	M	Moderate	A+T+(N)+/ AD
6	65	M	Moderate	A+T+(N)+ / AD
7	61	F	Mild	A+T+(N)+ /AD
8	74	M	Severe	Alzheimer's clinical syndrome without biomarkers tested
9	74	F	Moderate	A+T+(N)+ /AD
10	76	M	Mild	A-T-(N)+^b^
11	72	M	Mild	A+T+(N)+ / AD

Cognitive impairment was defined using the Mini-Mental Status Examination (MMSE): 30–27, no impairment, 26–20, mild impairment, 19–10, moderate impairment, and <10, severe impairment. Diagnostic criteria were assessed according to the NIA-AA criteria. “A” labels biomarker of Aß plaques, “T” labels biomarkers of fibrillar tau, and “N” labels biomarkers of neurodegeneration or neuronal injury (10). Two patients were included with no biomarkers tested.

a Alzheimer's and concomitant suspected non-Alzheimer's pathological change.

b Alzheimer's clinical syndrome with non-Alzheimer's pathological change.

The exclusion criteria for TPS treatment were relevant intracerebral pathologies (including vascular lesions Fazekas > 2) unrelated to Alzheimer's disease, non-compliance with the protocol, blood clotting disorders, oral anticoagulation, corticosteroid treatment in the last 6 weeks, pregnancy, breastfeeding, or epilepsy. Patients signed informed consent to receive the stimulation treatment. The retrospective analysis of all patients treated with TPS was part of the local registry approved by the Ethics Committee of the regional Medical Chamber (Ärztekammer Nordrhein, Nr. 2021026). Patients varied in the severity of cognitive symptoms: four patients with mild, five with moderate, and two with severe impairment (see Table 1).

### Materials

Numerous cognitive and affective scores were assessed as part of the standard assessment before the first stimulation and on the last day of the stimulation protocol.

#### Mini-mental status examination

The MMSE tests orientation, word recall, attention, language abilities, calculations, and visuospatial ability. This test was conducted before the first treatment and after the last treatment, and it was used as a screening tool for the classification of symptom severity. A heterogeneous group that was defined in its symptom severity using the Mini-Mental Status Examination (MMSE) with a range from 2 to 27 (*M* = 17.64, SD = 7.74) was treated and thus included in the database average of 17.64 (SD = 7.74).

#### Alzheimer's disease assessment scale

The ADAS total score, including the ADAS cognitive score (ADAS Cog), were used as a parameter for follow-up on the cognition of the patients. The noncognitive subscale includes interviews with the patient on the mood and behavior changes. The cognitive subscale includes 11 tasks with subject-completed tests and observations from the neuropsychologist. The ADAS test takes about 45 min, and the patient's performance is ranked with a score from 0 to 150 by summing the number of errors made on each task. Therefore, the lower the score, the better the performance of the patient. After the last TPS treatment, a parallel version was administered.

#### Montreal cognitive assessment

The MoCA assesses the same areas as the MMSE but provides more depth and includes additional cognitive functions measured with a clock-drawing test and a trail-making test. A parallel version of this test was administered after the TPS treatment.

#### Numeric rating scale

The severity of the symptoms as well as side effects were assessed using the NRS. This scale ranges from 0 to 10, with higher numbers indicating higher intensity. Before each treatment, the patient was asked if they experienced any side effects and to subjectively evaluate the severity of their main symptom during the last 24 h. If the patient was not able to answer the question due to the severity of the disease, caregivers were questioned.

### Stimulation

**Figure 1:**
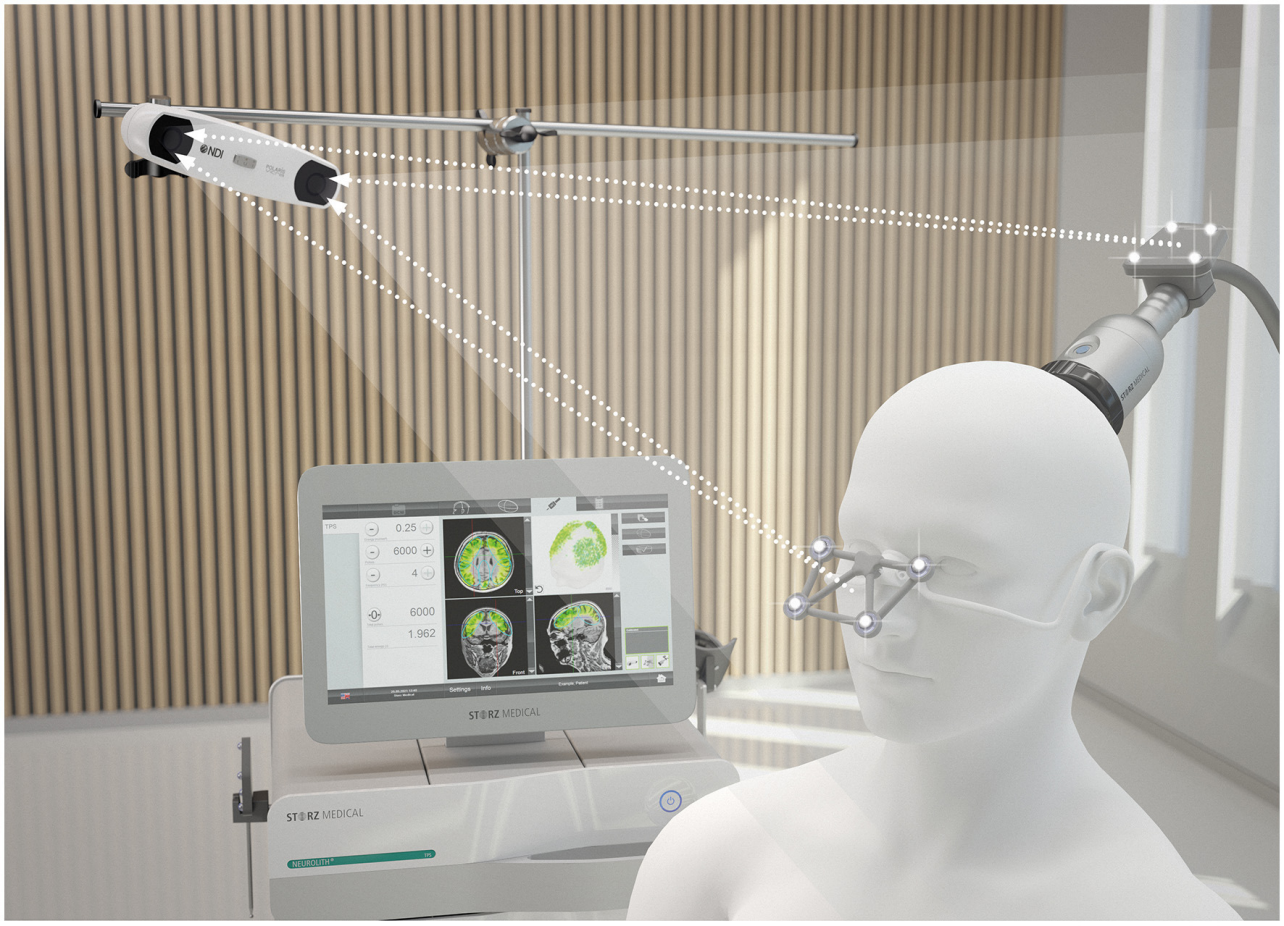
TPS system. 3D camera, navigation tracker and headpiece, and TPS handpiece with naviagation tracker. Image Source: Storz Medical.

**Figure 2:**
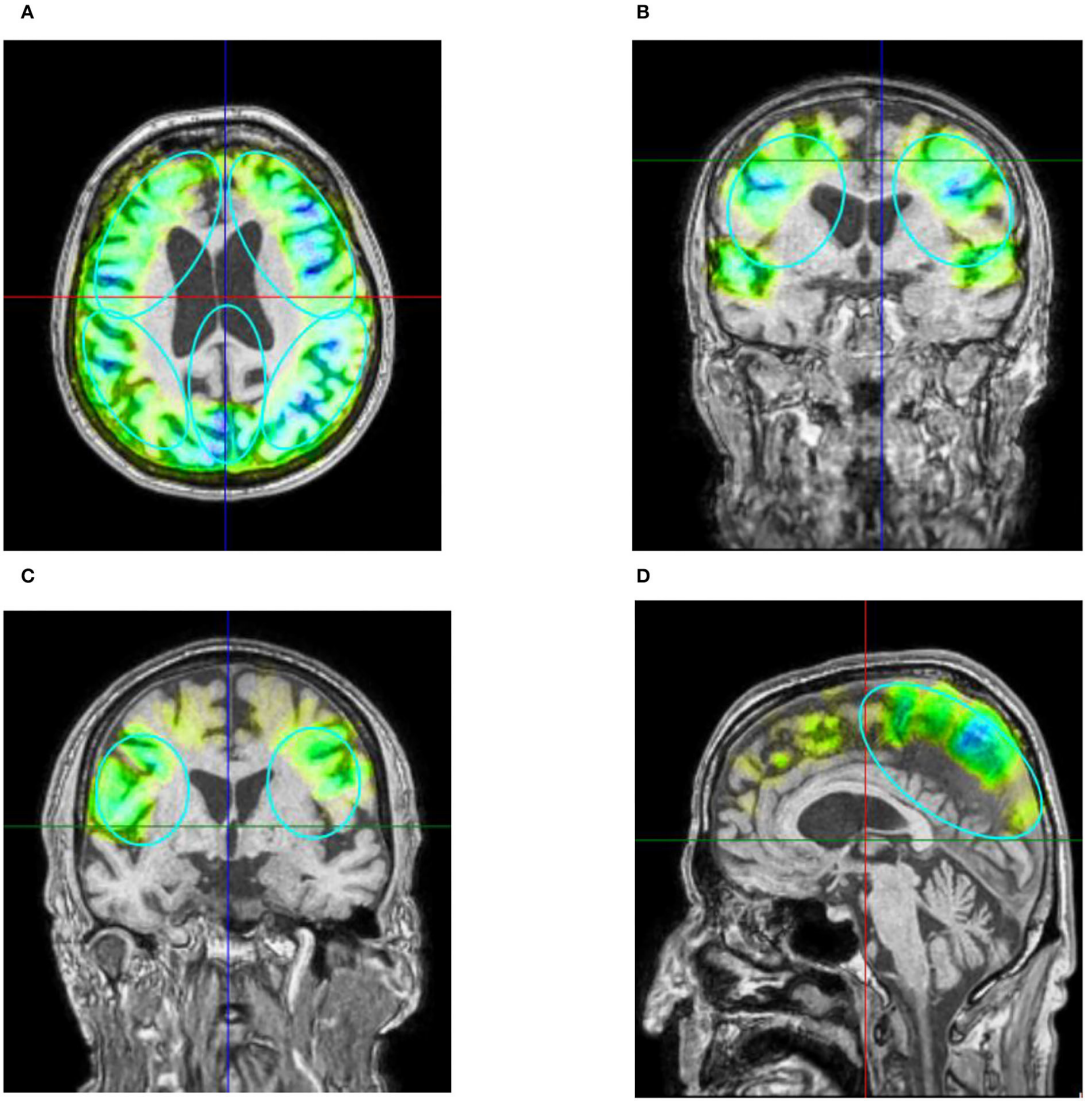
**(A)** Example of axial T1 image with navigated visualization of applied pulse energy. Color code shows quantity of pulses and energy applied in predefined ROIs (turquoise ellipses) frontal, parietal, and precuneus with green indicating low, yellow indicating medium, and blue indicating high energy applied. Image source: Storz Medical. **(B)** Coronar T1 image of patient 10. Besides predefined ROI, pulses were also applied to the temporal cortex perinsular. **(C)** The parietal treatment ROI on coronar T1 from patient 2. **(D)** The treatment of precuneus with 600 pulses visualized on ROI on sagittal T1 image of patient 3. Please note that 3D ROIs are partly superimposed from other 2D plane sections.

For the stimulation, the Neurolith© TPS device from Storz Medical was used, which allows neuronavigation using individual 3D T1 isometric voxel MRI scans (Figure 1). The treatment protocol was 4 Hz, 0.20 mJ/mm^2^ by default. The stimulated areas were similar to Beisteiner et al. (7), including the bilateral frontal cortex, bilateral lateral parietal cortex, and extended precuneus cortex (Figure 2A). Yet, the bilateral temporal cortex was also stimulated (Figure 2B). Treatment protocol was either in six sessions with 6,000 pulses over 2 weeks with a ≥48 h break between sessions or in 12 sessions with 3,000 pulses every day.

### Statistical analysis

Several hypotheses were addressed: For hypothesis I, NRS scales were descriptively analyzed. For hypothesis II, NRS scales were also analyzed using a one sided *t*-test with an alpha = 0.05 for significance. Hypothesis III was tested using the changes in ADAS total score, ADAS-Cog, MMSE, and MoCA from the baseline to the follow-up assessment and by computing a one-sided *t*-test with alpha = 0.05 for significance. For testing hypothesis IV, patients were divided into three groups, namely, mild cognitive impairment (*N* = 4), moderate cognitive impairment (*N* = 5), and severe cognitive impairment (*N* = 2) using the MMSE cutoff criteria and compared descriptively. Furthermore, a one-way spearman's rank correlation between MMSE scores from the baseline testing and the improvement in each test after stimulation was calculated. For all analyses, SPSS Version 27.0.1.0 and Microsoft Excel were used.

## Results

### Side effects

Notably, three out of 11 patients (27%) reported side effects in three out of 75 total sessions (4%). These included pain in the jaw (NRS 4/10), feeling of nausea (NRS 7/10), and drowsiness (NRS 10/10). Medical assessments (blood count, blood sugar, and blood pressure) could not reveal the cause of the drowsiness of one patient, and external reasons could not be ruled out. None of the side effects lasted longer than one day, and, therefore, no permanent side effects were observed.

### Subjective improvement of symptom severity

Descriptive analysis showed a large individual variation in estimating the improvement. Four of the patients were not able to evaluate the severity of their symptoms. Of the seven patients, six reported an improvement. The mean subjective improvement of the symptom severity (*N* = 7) in NRS was from 5.7 (*SD* = 2.3) to 3.4 (*SD* = 3) [*t*_(5)_ = 2.65, *p* = 0.023]. Moreover, a one-tailed *t*-test reveal a significant difference in the depressive symptoms in a self-reported subscale of the ADAS test before (*M* = 0.7, *SD* = 1.1) and after stimulation (*M* = 0.2, *SD* = 0.4) [*t*_(8)_ = 1.859, *p* < 0.01].

### Short-term effects on cognitive functions

**Figure 3:**
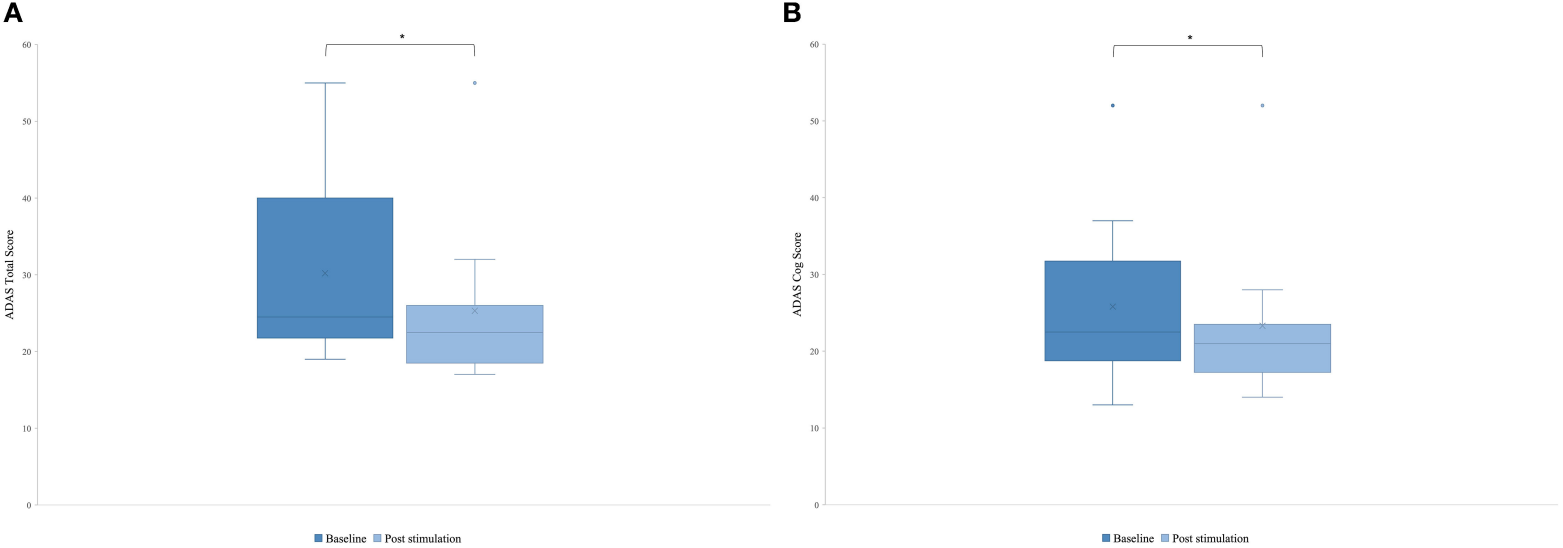
Mean of the patient group's score of the Alzheimer's Disease Assessment Scale (ADAS) before the first stimulation (dark blue) and after the last stimulation (light blue). A lower score indicates a better performance. Box plot show destribution of the patients' data. **(A)** ADAS total score. The line represents the median of the group (*baseline* = 24.5, *post-stimulation* = 22.5), and the cross represents the mean scores [*M baseline* = 30.2 (*SD* 11.55), *M post-stimulation* = 25.8 (*SD* 10.71), *p = 0.01]. **(B)** ADAS cog score. The line represents the median of the group (*baseline* =22.5, *post-stimulation* = 21), and the cross represents the mean scores [*M baseline* = 25.8 (*SD* 10.77), *M post-stimulation* = 23.3 (*SD* 10.27), *p = *0.0*4].

There was a significant difference in the post-stimulation compared to the baseline in the ADAS total score (Figure 3A) and in the ADAS Cog score (Figure 3B). Means were for ADAS total score of 30.2 (SD = 11.55) and 25.8 (SD = 10.71) with *t*_(8)_ = 2.87 and *p* = 0.01 and for ADAS Cog 25.8 (SD = 10.77) and 23.3 (SD = 10.27) with *t*_(8)_ = 2, and *p* = 0.04, giving a total improvement in the ADAS total score of 15.76% and in the ADAS Cog score of 8.65%.

**Figure 4:**
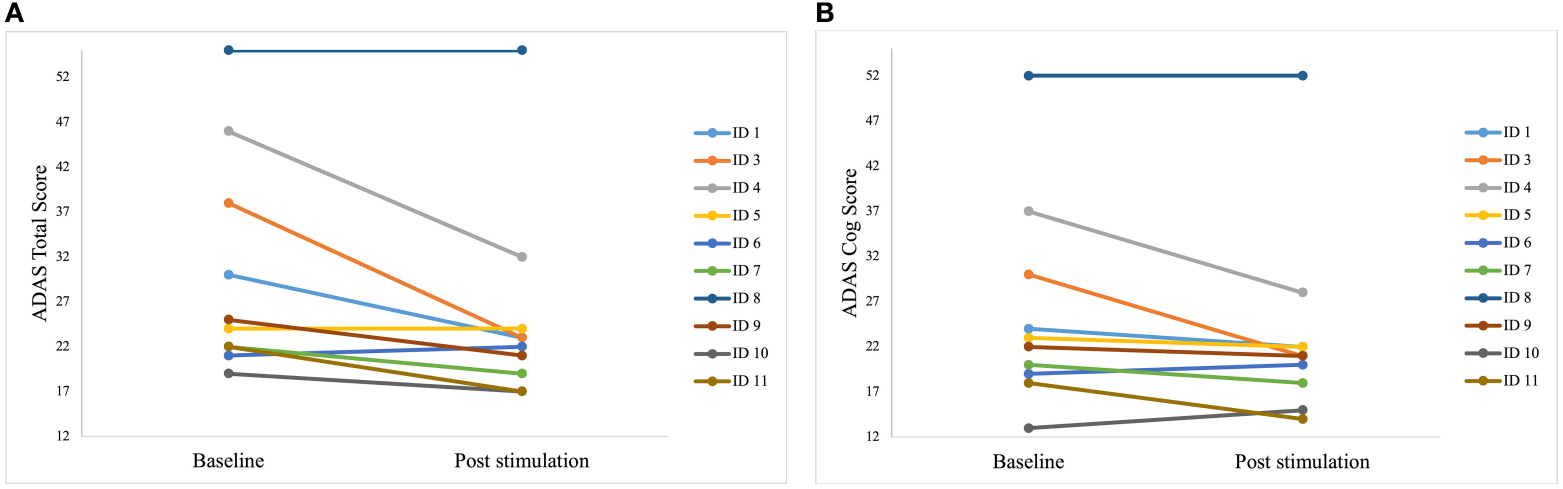
Individual test results of the patients in Alzheimer's Disease Assessment Scale (ADAS) before the first stimulation (*baseline*) and after the last stimulation (*post-stimulation*). A lower score indicates a better performance. Each line represents one patient. **(A)** Individual scores of each patient in the ADAS total score. Best improvement was 15 points (*ID 3*). **(B)** Individual scores of each patient in the sub scale ADAS cog score. Best improvement was 14 points (*ID 3* and *ID 4*).

Some patients only showed minor improvements, but the best improvement in a patient was 40% (Figure 4).

However, no significant difference was found neither in the MMSE with means of 17.64 (SD = 7.74) and 18 (SD = 7.12) with *t*_(9)_ = −0.80, *p* = 0.22, nor in the MoCA with means of 11.73 (SD = 6.2) and 12.09 [SD = 6.68 with *t*_(9)_ = −0.13, *p* = 0.45].

### Effectiveness between groups

The descriptive analysis of the different groups revealed a large improvement in the severe group (MMSE <10) and the moderate group (MMSE 19–10) in the MMSE. When looking at the patients with severe AD (MMSE <10), the mean score of the MMSE improved by 20% (*M improvement* = 0.55). Patients with mild symptoms (MMSE >20) worsened slightly (Table 2). In all tests, the moderate group improved more than the mild cognitive impairment group. In MoCA, the severe group (MMSE <10) worsened, while the moderate group and the mild group improved.

**Table 2:** Normalized absolute and relative mean change of the scores for the different groups: Mild cognitive impairment, moderate cognitive impairment, and severe cognitive impairment.

	**Mild (*N* = 4)**	**Moderate (*N* = 5)**	**Severe (*N* = 2)**
MMSE	−0.75 (−2.91%)	+1.4 (8.64%)	+0.55 (20%)
ADAS total	+4.25 (18.28%)	+6.4 (20.78%)	--^*^
ADAS Cog	+1.5 (8%)	+3.8 (14.5%)	-^*^
MoCA	+0.25 (3.83%)	+1.6 (15.69%)	−1.5 (−60%)

Positive values indicate improvement and negatives values indicate worsening.

* For ADAS, the severe cognitive impairment group was N = 1.

The statistical test of hypothesis IV–if TPS effects differed between mildly, moderate, or severely patients–however, showed no significant correlation between baseline MMSE and changes of cognitive scores after treatment.

Yet the MMSE showed a negative and moderate correlation with ρ = −0.436, and the ADAS total score showed a moderate and positive correlation with ρ = 0.396. Weak correlations were found in the ADAS Cog with ρ = 0.12 and MoCA with ρ = 0.063.

The three non-diagnosed AD patients (two patients without biomarkers tested and one with Alzheimer's clinical syndrome with non-diagnosed Alzheimer's pathological change) did show cognitive changes after stimulation in a comparable range as did the AD group (*N* = 8) in most tests. Due to the small sample size, we did not apply statistics. In detail, the mean numbers were as follows:

ADAS total score (non-diagnosed AD group: M improvement = 5.67; AD group: M improvement = 4) and ADAS Cog (non-diagnosed AD group: M improvement = 2.33; AD group: M improvement = 2.2).

MMSE (non-diagnosed AD group: M improvement = 1.67; AD group: worsened slightly, M improvement = −0.13), MoCA (non-diagnosed AD group: M improvement = 1; AD group: M improvement = 0.125).

## Discussion

This research implied a retrospective investigation of TPS in a heterogeneous sample of patients with AD and tested several hypotheses: (I) side effects occurred rarely, which indicates that TPS is a safe and in general well tolerated; (II) the subjective reports of the improvement of the main symptom exposed a significant effect after the treatment. Also, a significant difference was found in the depressive symptoms measured by a self-reported ADAS subscale; (III) these preliminary results display a significant cognitive improvement in patients after TPS treatment in the ADAS total score and the ADAS Cog; and (IV) no significant difference in improvement according to baseline symptom severity was proven. However, between the groups' mild cognitive impairment, moderate cognitive impairment, and severe cognitive impairment, a slight difference was suggested by the data: descriptive analysis of the data indicates a larger improvement in severe and moderately affected patient compared to mildly affected patients in most tests. Our findings suggest that severe and moderately affected patients at least benefit equally from TPS as mildly affected patients, but a ceiling effect in mildly affected patients must be considered. Moreover, two of the patients were identified as having Alzheimer's clinical syndrome without having biomarkers tested, and one showed an Alzheimer's clinical syndrome with non-diagnosed Alzheimer's pathological change. This subgroup's mean scores also improved cognitively after stimulation, indicating that no pathological AD diagnosis is needed to find an improvement after stimulation. Yet, it must be considered that the sample size is not representative enough and the difference in scores between subgroups might be due to the small sample size (*N* = 3) and the fact that the non-diagnosed AD group included two moderate/severe cognitive impairment patients as possible bias. Furthermore, the improvements in cognition varied between the different neuropsychological tests, which could be explained by the different sensitivities of the assessments. The Alzheimer's Disease Assessment Scale (ADAS), which is used in this study, has been conducted to assess the effects of anti-dementia treatments since 1980 (12). It has been developed to evaluate the severity of cognitive and non-cognitive deficits from mild to severe AD. However, the ADAS test has been criticized for not being able to detect changes at milder stages of Alzheimer's disease (12). The MoCA was developed to detect earlier stages of dementia and is commonly used for mild cognitive impairment (MCI) since it is more challenging than other dementia tests (13). The MMSE was also developed to detect MCI, yet it is less sensitive due to its lack of complexity and absence of executive function items (14). These differences in the sensitivity of the tests between the stages of AD could also explain the different results of this study within the tests. The total patient group showed a significant cognitive improvement in ADAS total and ADAS Cog, but not in the MMSE or MoCA. This is underlined by the fact that the group sample used for this study was more advanced in the symtopms, which the ADAS test is more sensitive to than the other tests.

Additionally, the patients reported a significant improvement in subjective symptom severity. However, the scores showed a large individual variation regarding the change, which might be caused by a placebo effect. This is the first demonstration of improving cognition in patients with severe Alzheimer's disease using TPS; however, there are limitations to be considered. First, there was no sham stimulation as a control condition. Second, the sample size is small. Due to the limitations of a retrospective analysis, data were entered in a clinical database and not collected for research, which caused some missing data from patients in some tests. In conclusion, TPS can be included as an effective and safe add-on treatment for Alzheimer's disease. Even though the first studies (7, 9, 10), as well as our findings, show promising results, more research is needed, including long-term results in patients. Besides larger sham controlled trials, translational research on the mechanisms of action and effects on cerebral network physiology will be needed. Vascular, metabolic, neurotrophic, and (meta-)plasticity effects will need to be investigated (15).

## Data availability statement

The raw data supporting the conclusions of this article will be made available by the authors, without undue reservation.

## Ethics statement

The registry involving human participants was reviewed and approved by Ärztekammer Nordrhein, Nr. 2021026. Patients gave written informed consent to the treatment and consent to be included in the registry. Registry data and images have been anonymized.

## Author contributions

CC, NS, AG, CS, KL, CT, and LW contributed to conception and design of the study. CC, NS, AG, CS, KL, and LW did the data acquisition. NS, AG, CS, and CC organized the database. CC performed the statistical analysis and wrote the first draft of the manuscript. LW wrote sections of the manuscript. All authors contributed to manuscript revision, read, and approved the submitted version.

## Funding

This study received funding from the Storz Medical for payment of publication fees and conference costs for data presentation. The funder was not involved in the study design, collection, analysis, interpretation of data, the writing of this article, or the decision to submit it for publication.

## Conflict of interest

Author CC received travel fees from Storz Medical. LW has previously received funding grants and institutional support from the German Research Foundation, Hilde-Ulrichs-Stiftung für Parkinsonforschung, and the ParkinsonFonds Germany, BMBF/ERA-NETNEURON, DFG Forschergruppe (FOR1328), Deutsche Parkinson Vereinigung (DPV), Forschungskommission, Medizinische Fakultät, HHU Düsseldorf, UCB; Medtronic, UCB, Teva, Allergan, Merz, Abbvie, Roche, Bial, Merck, Novartis, Desitin, Spectrum. LW owned stock in company BioNTech SE. He is consultant to the following companies or received travel honarium from: TEVA, UCB Schwarz, Desitin, Medtronic, Abbott/Abbvie, MEDA, Boehringer I, Storz Medical, Kyowa Kirin, Guidepoint, Merck, Merz, Synergia, BIAL, Zambon, Sapio Life, STADA, Inomed, Vertanical. The remaining authors declare that the research was conducted in the absence of any commercial or financial relationships that could be construed as a potential conflict of interest.

## Publisher's note

All claims expressed in this article are solely those of the authors and do not necessarily represent those of their affiliated organizations, or those of the publisher, the editors and the reviewers. Any product that may be evaluated in this article, or claim that may be made by its manufacturer, is not guaranteed or endorsed by the publisher.
